# Crystal structure of [Ag(NH_3_)_3_]_2_[Ag(NH_3_)_2_]_2_[SnF_6_]F_2_, a compound showing argentophilic inter­actions

**DOI:** 10.1107/S2056989016019010

**Published:** 2016-11-29

**Authors:** Florian Kraus, Matthias Fichtl, Sebastian Baer

**Affiliations:** a Anorganische Chemie, Fluorchemie, Fachbereich Chemie, Philipps-Universität Marburg, Hans-Meerwein-Strasse 4, 35032 Marburg, Germany

**Keywords:** crystal structure, silver, argentophilic inter­actions, fluorides, ammine ligand

## Abstract

Bis[triamminesilver(I)] bis­[diamminesilver(I)] hexa­fluorido­stannate(IV) difluoride, [Ag(NH_3_)_3_]_2_[Ag(NH_3_)_2_]_2_[SnF_6_]F_2_, was obtained in the form of colourless crystals from the reaction of CsAgSnF_7_ in anhydrous ammonia. Two different ammine complexes of silver(I) are present in the structure, *i.e.* a linear diammine and a T-shaped triammine complex. The ammine silver(I) complexes show Ag⋯Ag distances in the range of argentophilic inter­actions. In the crystal, several N—H⋯F hydrogen bonds are present between the complex cations and the SbF_6_^−^ and F^−^ anions, leading to the formation of a three-dimensional network.

## Abstract 2

The structure of [Ag(NH_3_)_3_]_2_[Ag(NH_3_)_2_]_2_[SnF_6_]F_2_ contains linear diammine silver(I) and T-shaped triammine silver(I) cations which show short Ag⋯Ag distances in the range of argentophilic inter­actions.

## Chemical context   

Metallophilicity, especially argento- and aurophilicity, is a theoretically and experimentally well-established concept, see, for example, the seminal works of Jansen (Jansen, 1987 ▸), Schmidbaur and co-workers (Scherbaum *et al.*, 1988 ▸; Schmidbaur, 1995 ▸; Schmidbaur & Schier, 2012 ▸, 2015 ▸) or Pyykkö and co-workers (Pyykkö & Zhao, 1991 ▸; Pyykkö, 1997 ▸,2004 ▸; Pyykkö *et al.*, 1997 ▸; Pyykkö & Mendizabal, 1997 ▸). We reacted a silver(II) compound, CsAgSnF_7_, with anhydrous liquid ammonia and observed the reduction of Ag^II^. The preparation conditions and crystal structure of the thus obtained Ag^I^ title compound, [Ag(NH_3_)_3_]_2_[Ag(NH_3_)_2_]_2_[SnF_6_]F_2_, is reported here. The short Ag⋯Ag distances between the complex cations are in the range of argentophilic inter­actions.

## Structural commentary   

**Figure 1:**
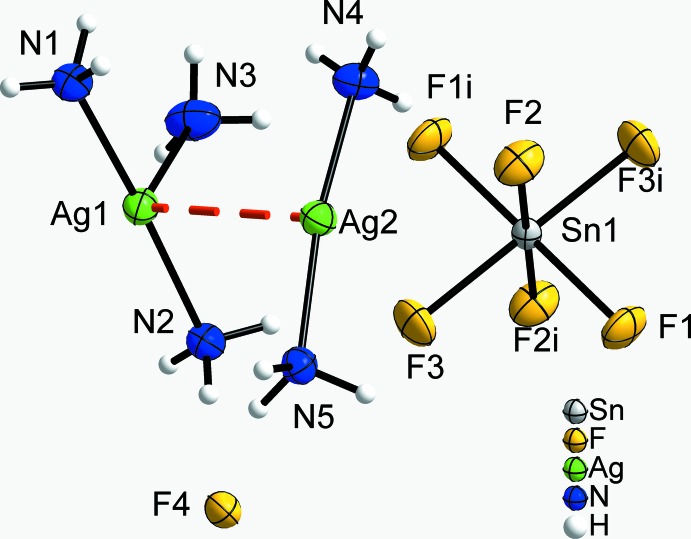
The principal building units in the crystal structure of the title compound, showing the F^−^ anion, the [SnF_6_]^2−^ anion, as well as the argentophilic inter­action (in red) between the [Ag(NH_3_)_2_]^+^ and [Ag(NH_3_)_3_]^+^ cations. Displacement ellipsoids are drawn at the 70% probability level and H atoms are shown with an arbitrary radius. [Symmetry code: (i) −*x* + 1, −*y*, −*z* + 1.]

[Ag(NH_3_)_3_]_2_[Ag(NH_3_)_2_]_2_[SnF_6_]F_2_ crystallizes in space group type *P*2_1_/*c*. The Sn atom occupies Wyckoff position 2*d* (site symmetry 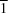), all other atoms reside on general positions 4*e*. The structure comprises of [Ag(NH_3_)_3_]^+^ and [Ag(NH_3_)_2_]^+^ complex cations as well as F^−^ and [SnF_6_]^2−^ anions (Fig. 1 ▸). The diamminesilver(I) cation (Ag2) is almost linear with an N—Ag—N angle of 170.93 (7)° and Ag—N distances of 2.1160 (16) and 2.1183 (16) Å. The deviation from linearity is likely to arise from the surrounding, *i.e.* N—H⋯F hydrogen bonding to adjacent [SnF_6_]^2−^ and F^−^ anions. This [Ag(NH_3_)_2_]^+^ cation shows a short Ag⋯Ag distance of 3.0611 (2) Å to a neighboring [Ag(NH_3_)_3_]^+^ cation and another slightly longer Ag⋯Ag distance of 3.3282 (2) Å to a second [Ag(NH_3_)_3_]^+^ cation (symmetry code *x*, −*y* + 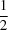, *z* + 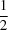). The triammine silver(I) cation (Ag1) is T-shaped and can be viewed as a linear diammine silver(I) cation to which another ammine ligands is bound at a longer distance. The short Ag—N distances are 2.1434 (16) and 2.1662 (16) Å, and the remote ammine ligand is bound at a distance of 2.5870 (19) Å. The N—Ag—N angle between the shortly bonded ligands is 173.74 (7)°. The deviation of N—Ag—N angles including the remote ammine ligand from 90° [85.44 (6) and 110.82 (6)°] are probably due to hydrogen bonding of the ammine ligands to F atoms of the anions.

## Supra­molecular features   

**Figure 2:**
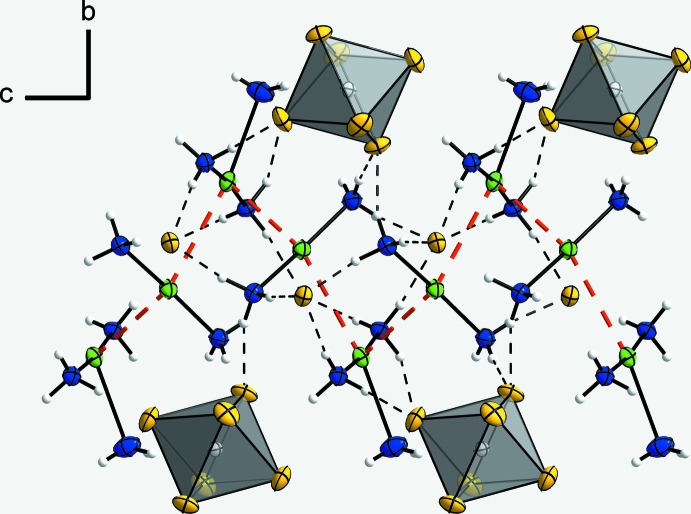
A section of the crystal structure in a view along [100], showing a corrugated strand of complex cations running along [001]. The argentophilic inter­actions are drawn as red dashed bonds between the Ag^I^ atoms and N—H⋯F hydrogen bonds are shown as dashed lines. [SnF_6_]^2−^ anions are shown as polyhedra to highlight their positions relative to the the kinks of the strand. Displacement ellipsoids are as in Fig. 1 ▸.

As a result of the short Ag1⋯Ag2 contacts, corrugated strands of alternating [Ag(NH_3_)_3_]^+^ and [Ag(NH_3_)_2_]^+^ cations occur where the [Ag(NH_3_)_3_]^+^ cations form the kinks which are connected by the [Ag(NH_3_)_2_]^+^ cations. The strands run parallel to the *c* axis (Fig. 2 ▸). Similar metallophilic inter­actions have been observed in the ammine copper(I) fluoride {[Cu(NH_3_)_3_]_2_[Cu_2_(NH_3_)_4_]}F_4_·4NH_3_ (Woidy *et al.*, 2015 ▸). However, the cuprophilic inter­actions are only observed between the diammine copper(I) cations forming linear strands whereas the triammine copper(I) cations do not show such inter­actions.

**Figure 3:**
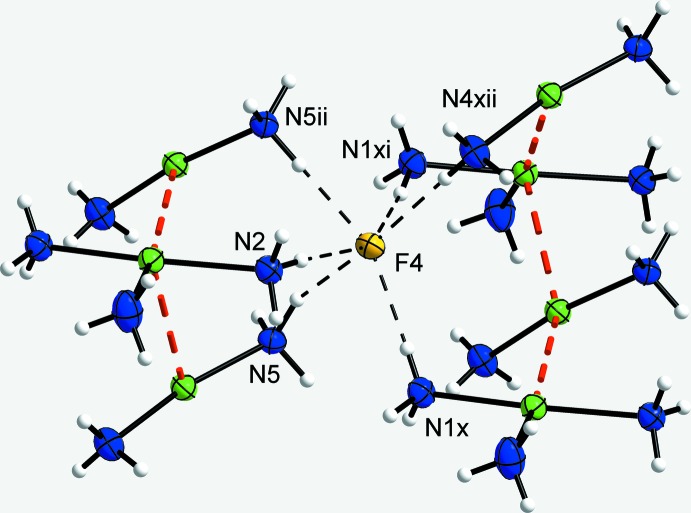
A section of the crystal structure of the title compound, showing the N—H⋯F hydrogen bonds (dashed lines) around the free fluoride anion and the bridging of the corrugated Ag⋯Ag strands (red dashed lines). Displacement ellipsoids are as in Fig. 1 ▸. [Symmetry codes: (ii) *x*, −*y* + 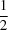, *z* + 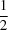; (*x*) 1 + *x*, *y*, *z*; (xi) 1 + *x*, 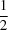 − *y*, 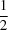 + *z*; (xii) 1 + *x*, *y*, 1 + *z*.]

**Figure 4:**
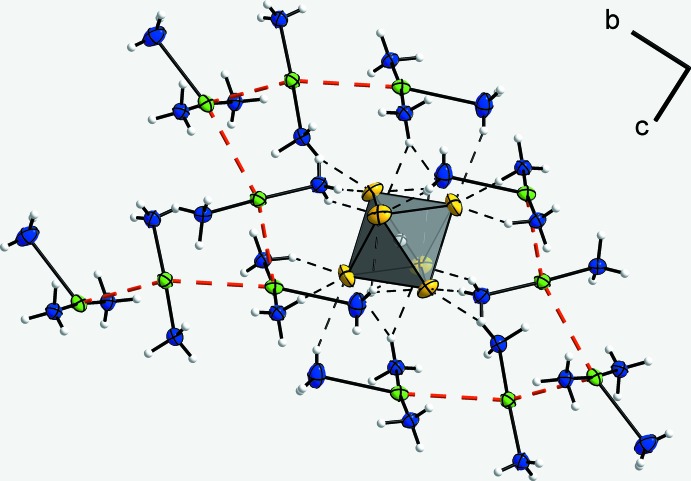
A section of the crystal structure, showing the hydrogen bonding towards the [SnF_6_]^2−^ anion, which is shown as a polyhedron. Ag^I^ atoms are inter­connected by Ag⋯Ag inter­actions (red dashed lines) to show the formation of strands. Displacement ellipsoids are as in Fig. 1 ▸.

**Figure 5:**
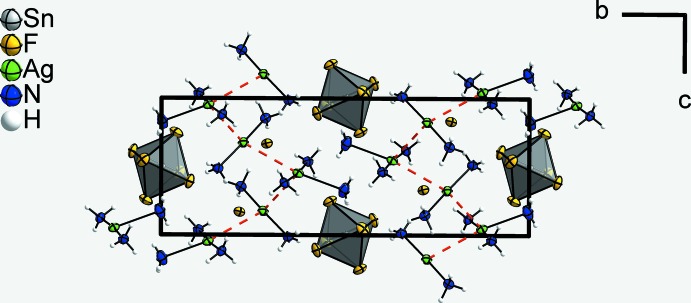
The crystal structure of the title compound. Ag^I^ atoms are inter­connected by argentophilic inter­actions (red dashed lines) to show the formation of strands and [SnF_6_]^2−^ anions are shown as polyhedra. Displacement ellipsoids are as in Fig. 1 ▸.

In the title structure, the fluoride anions reside above and below the cation strands and connect neighbouring strands *via* N—H⋯F hydrogen bonds, whereas the [SnF_6_]^2−^ anions lie on the sides of the strands, also connecting neighbouring ones. The free fluoride ion (F4) is an acceptor of six hydrogen bonds (Fig. 3 ▸). Its coordination environment resembles an octa­hedron with one longer edge. It inter­connects the Ag⋯Ag strands along the *a*-axis. The [SnF_6_]^2−^ anion inter­connects four of the Ag⋯Ag strands (Fig. 4 ▸). Four of the six F atoms bonded to the Sn atom are acceptors of four hydrogen bonds (two regular, two bifurcated), the other two F atoms are acceptors of three hydrogen bonds. The diammine silver(I) cations only form regular hydrogen bonds, whereas the triammine silver(I) cations form regular as well as bifurcated hydrogen bonds. The bifurcated hydrogen bonds bridge four edges of each [SnF_6_]^2−^ octa­hedron. Overall, a rather complex three-dimensional hydrogen-bonded network results (Fig. 5 ▸). Numerical details of the hydrogen-bonding inter­actions are summarized in Table 1 ▸.

## Synthesis and crystallization   

870 mg of CsAgSnF_7_ were reacted with approximately 10 ml of anhydrous liquid ammonia at 195 K. Upon contact, the greenish colour of the educt vanished and a white powder was observed. This indicates that Ag^II^ was reduced to Ag^I^ and ammonia was oxidized to N_2_. From this white powder, colorless crystals grew within three months of storage at 233 K of which a suitable one was selected for the diffraction experiment. The role of the Cs atoms remains unclear.

## Refinement   

Crystal data, data collection and structure refinement details are summarized in Table 2 ▸. Hydrogen atoms were localized from difference Fourier syntheses and were refined freely.

## Supplementary Material

Crystal structure: contains datablock(s) I. DOI: 10.1107/S2056989016019010/wm5342sup1.cif

Structure factors: contains datablock(s) I. DOI: 10.1107/S2056989016019010/wm5342Isup2.hkl

CCDC reference: 1519625

Additional supporting information: crystallographic information; 3D view; checkCIF report

## Figures and Tables

**Table 1:** Hydrogen-bond geometry (Å, °)

*D*—H⋯*A*	*D*—H	H⋯*A*	*D*⋯*A*	*D*—H⋯*A*
N1—H1*A*⋯F4^i^	0.85 (3)	2.03 (3)	2.884 (2)	178 (2)
N1—H1*B*⋯F4^ii^	0.89 (3)	1.96 (3)	2.844 (2)	171 (3)
N1—H1*C*⋯F3^i^	0.98 (4)	2.14 (4)	3.057 (2)	156 (3)
N2—H2*A*⋯F1^iii^	0.91 (3)	2.43 (3)	3.227 (2)	146 (2)
N2—H2*A*⋯F3^iv^	0.91 (3)	2.56 (3)	3.354 (2)	145.4 (19)
N2—H2*B*⋯F3	0.94 (3)	2.04 (3)	2.961 (2)	167 (3)
N2—H2*C*⋯F4	0.83 (3)	2.02 (3)	2.849 (2)	172 (3)
N3—H3*A*⋯F1^i^	0.88 (3)	2.57 (3)	3.274 (2)	138 (3)
N3—H3*A*⋯F2^v^	0.88 (3)	2.42 (3)	3.223 (2)	153 (3)
N3—H3*B*⋯F3^iv^	0.79 (3)	2.61 (3)	3.345 (3)	157 (3)
N3—H3*C*⋯F1^vi^	0.91 (3)	2.39 (3)	3.279 (3)	167 (3)
N4—H4*A*⋯F2	0.90 (3)	2.04 (3)	2.930 (2)	170 (2)
N4—H4*B*⋯F4^vii^	0.84 (3)	1.97 (3)	2.7955 (19)	171 (3)
N4—H4*C*⋯F1^i^	0.79 (3)	2.33 (3)	3.045 (2)	151 (3)
N5—H5*A*⋯F4	0.96 (3)	1.90 (3)	2.8305 (19)	160 (3)
N5—H5*B*⋯F4^viii^	0.95 (3)	1.93 (3)	2.882 (2)	173 (2)
N5—H5*C*⋯F2^ix^	0.93 (3)	2.09 (3)	2.999 (2)	163 (3)

Symmetry codes: (i) 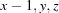; (ii) 
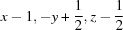
; (iii) 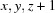; (iv) 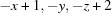; (v) 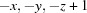; (vi) 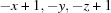; (vii) 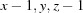; (viii) 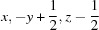; (ix) 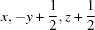.

**Table 2:** Experimental details

Crystal data	
Chemical formula	[Ag(NH_3_)_3_]_2_[Ag(NH_3_)_2_]_2_[SnF_6_]F_2_
*M*_r_	872.51
Crystal system, space group	Monoclinic, *P*2_1_/*c*
Temperature (K)	123
*a*, *b*, *c* (Å)	7.3274 (2), 19.4495 (4), 7.8579 (3)
β (°)	113.205 (4)
*V* (Å^3^)	1029.27 (6)
*Z*	2
Radiation type	Mo *K*α
μ (mm^−1^)	5.01
Crystal size (mm)	0.20 × 0.05 × 0.05

Data collection	
Diffractometer	Oxford-Diffraction Xcalibur3
Absorption correction	Multi-scan (*CrysAlis RED*; Oxford Diffraction, 2008 ▸)
*T*_min_, *T*_max_	0.602, 1.000
No. of measured, independent and observed [*I* > 2σ(*I*)] reflections	30418, 5731, 4330
*R*_int_	0.030
(sin θ/λ)_max_ (Å^−1^)	0.889

Refinement	
*R*[*F* ^2^ > 2σ(*F* ^2^)], *wR*(*F* ^2^), *S*	0.020, 0.046, 0.99
No. of reflections	5731
No. of parameters	167
H-atom treatment	All H-atom parameters refined
Δρ_max_, Δρ_min_ (e Å^−3^)	1.04, −0.93

Computer programs: *CrysAlis CCD* and *CrysAlis RED* (Oxford Diffraction, 2008 ▸), *SHELXS97* (Sheldrick, 2008 ▸), *SHELXL2016* (Sheldrick, 2015 ▸), *SHELXLE* (Hübschle *et al.*, 2011 ▸), *DIAMOND* (Brandenburg, 2012 ▸) and *publCIF* (Westrip, 2010 ▸).

## supplementary crystallographic information

### Crystal data

**Table:**

[Ag(NH_3_)_3_]_2_[Ag(NH_3_)_2_]_2_[SnF_6_]F_2_	*F*(000) = 820
*M**_r_* = 872.51	*D*_x_ = 2.815 Mg m^−^^3^
Monoclinic, *P*2_1_/*c*	Mo *K*α radiation, λ = 0.71073 Å
*a* = 7.3274 (2) Å	Cell parameters from 16653 reflections
*b* = 19.4495 (4) Å	θ = 2.8–39.1°
*c* = 7.8579 (3) Å	µ = 5.01 mm^−^^1^
β = 113.205 (4)°	*T* = 123 K
*V* = 1029.27 (6) Å^3^	Block, colorless
*Z* = 2	0.20 × 0.05 × 0.05 mm

### Data collection

**Table:**

Oxford-Diffraction Xcalibur3 diffractometer	5731 independent reflections
Radiation source: Enhance (Mo) X-ray Source	4330 reflections with *I* > 2σ(*I*)
Graphite monochromator	*R*_int_ = 0.030
Detector resolution: 16.0238 pixels mm^-1^	θ_max_ = 39.2°, θ_min_ = 3.0°
phi– and ω–rotation scans	*h* = −12→13
Absorption correction: multi-scan (CrysAlis RED; Oxford Diffraction, 2008)	*k* = −34→30
*T*_min_ = 0.602, *T*_max_ = 1.000	*l* = −13→13
30418 measured reflections	

### Refinement

**Table:**

Refinement on *F*^2^	Secondary atom site location: difference Fourier map
Least-squares matrix: full	Hydrogen site location: difference Fourier map
*R*[*F*^2^ > 2σ(*F*^2^)] = 0.020	All H-atom parameters refined
*wR*(*F*^2^) = 0.046	*w* = 1/[σ^2^(*F*_o_^2^) + (0.0215*P*)^2^] where *P* = (*F*_o_^2^ + 2*F*_c_^2^)/3
*S* = 0.99	(Δ/σ)_max_ = 0.002
5731 reflections	Δρ_max_ = 1.04 e Å^−^^3^
167 parameters	Δρ_min_ = −0.93 e Å^−^^3^
0 restraints	Extinction correction: SHELXL2016 (Sheldrick, 2015), Fc^*^=kFc[1+0.001xFc^2^λ^3^/sin(2θ)]^-1/4^
Primary atom site location: structure-invariant direct methods	Extinction coefficient: 0.00277 (14)

### Special details

**Table:**

Geometry. All esds (except the esd in the dihedral angle between two l.s. planes) are estimated using the full covariance matrix. The cell esds are taken into account individually in the estimation of esds in distances, angles and torsion angles; correlations between esds in cell parameters are only used when they are defined by crystal symmetry. An approximate (isotropic) treatment of cell esds is used for estimating esds involving l.s. planes.

### Fractional atomic coordinates and isotropic or equivalent isotropic displacement parameters (Å^2^)

**Table:**

	*x*	*y*	*z*	*U*_iso_*/*U*_eq_	
SN1	0.500000	0.000000	0.500000	0.01140 (3)	
F1	0.71015 (17)	0.04893 (6)	0.45645 (18)	0.0254 (3)	
F2	0.31983 (17)	0.07728 (6)	0.39037 (17)	0.0232 (2)	
F3	0.58561 (19)	0.04104 (7)	0.74794 (16)	0.0274 (3)	
AG1	0.16018 (2)	0.12778 (2)	0.95234 (2)	0.01661 (3)	
N1	−0.1364 (2)	0.16467 (9)	0.8813 (2)	0.0179 (3)	
H1A	−0.170 (4)	0.1789 (14)	0.968 (4)	0.029 (7)*	
H1B	−0.171 (4)	0.2005 (14)	0.805 (4)	0.028 (7)*	
H1C	−0.222 (5)	0.1286 (19)	0.805 (5)	0.070 (11)*	
N2	0.4708 (2)	0.10144 (9)	1.0373 (2)	0.0175 (3)	
H2A	0.509 (4)	0.0701 (13)	1.131 (3)	0.021 (6)*	
H2B	0.485 (4)	0.0811 (15)	0.935 (4)	0.041 (8)*	
H2C	0.557 (5)	0.1316 (16)	1.084 (4)	0.044 (9)*	
N3	0.0911 (3)	0.00306 (10)	0.8291 (3)	0.0277 (4)	
H3A	−0.031 (5)	−0.0050 (15)	0.751 (5)	0.046 (9)*	
H3B	0.149 (5)	−0.0181 (16)	0.920 (5)	0.043 (9)*	
H3C	0.133 (5)	−0.0059 (15)	0.737 (4)	0.043 (9)*	
AG2	0.23394 (2)	0.22258 (2)	0.67497 (2)	0.01676 (3)	
N4	0.0292 (3)	0.15407 (9)	0.4859 (2)	0.0196 (3)	
H4A	0.111 (4)	0.1257 (14)	0.459 (4)	0.033 (7)*	
H4B	−0.052 (4)	0.1757 (14)	0.396 (4)	0.034 (7)*	
H4C	−0.034 (4)	0.1288 (15)	0.520 (4)	0.033 (8)*	
N5	0.4733 (2)	0.28292 (9)	0.8539 (2)	0.0167 (3)	
H5A	0.543 (4)	0.2617 (14)	0.973 (4)	0.033 (7)*	
H5B	0.562 (4)	0.2883 (12)	0.793 (3)	0.020 (6)*	
H5C	0.439 (5)	0.3248 (17)	0.891 (4)	0.052 (9)*	
F4	0.74284 (16)	0.21290 (6)	1.16824 (14)	0.0177 (2)	

### Atomic displacement parameters (Å^2^)

**Table:**

	*U*^11^	*U*^22^	*U*^33^	*U*^12^	*U*^13^	*U*^23^
SN1	0.01283 (6)	0.01077 (7)	0.01170 (7)	−0.00057 (5)	0.00600 (5)	−0.00041 (5)
F1	0.0194 (5)	0.0291 (7)	0.0314 (6)	−0.0056 (5)	0.0139 (5)	0.0066 (5)
F2	0.0207 (5)	0.0175 (5)	0.0319 (6)	0.0053 (4)	0.0110 (5)	0.0065 (5)
F3	0.0329 (6)	0.0314 (7)	0.0179 (5)	−0.0038 (5)	0.0098 (5)	−0.0095 (5)
AG1	0.01565 (5)	0.01867 (7)	0.01611 (6)	0.00126 (5)	0.00689 (4)	0.00184 (5)
N1	0.0179 (7)	0.0166 (7)	0.0183 (7)	0.0021 (6)	0.0061 (6)	0.0005 (6)
N2	0.0175 (6)	0.0178 (7)	0.0160 (7)	0.0000 (6)	0.0053 (5)	−0.0007 (6)
N3	0.0206 (8)	0.0236 (9)	0.0346 (10)	0.0001 (7)	0.0063 (8)	−0.0068 (8)
AG2	0.01614 (6)	0.01801 (7)	0.01510 (6)	−0.00111 (5)	0.00505 (4)	0.00066 (4)
N4	0.0184 (7)	0.0174 (8)	0.0194 (7)	−0.0010 (6)	0.0035 (6)	0.0012 (6)
N5	0.0169 (6)	0.0173 (7)	0.0161 (7)	0.0003 (5)	0.0066 (5)	0.0011 (5)
F4	0.0176 (5)	0.0200 (5)	0.0143 (5)	−0.0003 (4)	0.0051 (4)	−0.0015 (4)

### Geometric parameters (Å, º)

**Table:**

Sn1—F1^i^	1.9518 (11)	N2—H2A	0.91 (3)
Sn1—F1	1.9518 (11)	N2—H2B	0.94 (3)
Sn1—F2	1.9617 (11)	N2—H2C	0.83 (3)
Sn1—F2^i^	1.9617 (11)	N3—H3A	0.88 (3)
Sn1—F3^i^	1.9655 (11)	N3—H3B	0.79 (3)
Sn1—F3	1.9656 (11)	N3—H3C	0.91 (3)
Ag1—N1	2.1434 (16)	Ag2—N4	2.1160 (16)
Ag1—N2	2.1662 (16)	Ag2—N5	2.1183 (16)
Ag1—N3	2.5870 (19)	N4—H4A	0.90 (3)
Ag1—Ag2	3.0611 (2)	N4—H4B	0.84 (3)
Ag1—Ag2^ii^	3.3283 (2)	N4—H4C	0.79 (3)
N1—H1A	0.85 (3)	N5—H5A	0.96 (3)
N1—H1B	0.89 (3)	N5—H5B	0.95 (3)
N1—H1C	0.98 (4)	N5—H5C	0.93 (3)
			
F1^i^—Sn1—F1	180.0	Ag1—N2—H2A	110.8 (15)
F1^i^—Sn1—F2	90.56 (5)	Ag1—N2—H2B	107.2 (17)
F1—Sn1—F2	89.44 (5)	H2A—N2—H2B	108 (2)
F1^i^—Sn1—F2^i^	89.44 (5)	Ag1—N2—H2C	119 (2)
F1—Sn1—F2^i^	90.56 (5)	H2A—N2—H2C	100 (2)
F2—Sn1—F2^i^	180.0	H2B—N2—H2C	111 (3)
F1^i^—Sn1—F3^i^	90.58 (6)	Ag1—N3—H3A	115 (2)
F1—Sn1—F3^i^	89.42 (6)	Ag1—N3—H3B	101 (2)
F2—Sn1—F3^i^	88.80 (5)	H3A—N3—H3B	126 (3)
F2^i^—Sn1—F3^i^	91.19 (5)	Ag1—N3—H3C	113.7 (19)
F1^i^—Sn1—F3	89.42 (6)	H3A—N3—H3C	89 (3)
F1—Sn1—F3	90.58 (6)	H3B—N3—H3C	113 (3)
F2—Sn1—F3	91.19 (5)	N4—Ag2—N5	170.93 (7)
F2^i^—Sn1—F3	88.81 (5)	N4—Ag2—Ag1	81.11 (5)
F3^i^—Sn1—F3	180.0	N5—Ag2—Ag1	101.15 (5)
N1—Ag1—N2	173.74 (7)	N4—Ag2—Ag1^iii^	104.96 (5)
N1—Ag1—N3	100.82 (6)	N5—Ag2—Ag1^iii^	77.58 (4)
N2—Ag1—N3	85.44 (6)	Ag1—Ag2—Ag1^iii^	149.685 (7)
N1—Ag1—Ag2	93.35 (5)	Ag2—N4—H4A	101.3 (17)
N2—Ag1—Ag2	84.44 (5)	Ag2—N4—H4B	110.4 (19)
N3—Ag1—Ag2	111.20 (6)	H4A—N4—H4B	115 (3)
N1—Ag1—Ag2^ii^	77.42 (5)	Ag2—N4—H4C	120 (2)
N2—Ag1—Ag2^ii^	96.40 (5)	H4A—N4—H4C	104 (3)
N3—Ag1—Ag2^ii^	169.75 (6)	H4B—N4—H4C	107 (3)
Ag2—Ag1—Ag2^ii^	79.041 (4)	Ag2—N5—H5A	113.0 (16)
Ag1—N1—H1A	118.6 (17)	Ag2—N5—H5B	106.2 (14)
Ag1—N1—H1B	115.3 (18)	H5A—N5—H5B	109 (2)
H1A—N1—H1B	101 (2)	Ag2—N5—H5C	115.7 (19)
Ag1—N1—H1C	105 (2)	H5A—N5—H5C	100 (2)
H1A—N1—H1C	114 (3)	H5B—N5—H5C	112 (2)
H1B—N1—H1C	102 (3)		

Symmetry codes: (i) −*x*+1, −*y*, −*z*+1; (ii) *x*, −*y*+1/2, *z*+1/2; (iii) *x*, −*y*+1/2, *z*−1/2.

### Hydrogen-bond geometry (Å, º)

**Table:**

*D*—H···*A*	*D*—H	H···*A*	*D*···*A*	*D*—H···*A*
N1—H1*A*···F4^iv^	0.85 (3)	2.03 (3)	2.884 (2)	178 (2)
N1—H1*B*···F4^v^	0.89 (3)	1.96 (3)	2.844 (2)	171 (3)
N1—H1*C*···F3^iv^	0.98 (4)	2.14 (4)	3.057 (2)	156 (3)
N2—H2*A*···F1^vi^	0.91 (3)	2.43 (3)	3.227 (2)	146 (2)
N2—H2*A*···F3^vii^	0.91 (3)	2.56 (3)	3.354 (2)	145.4 (19)
N2—H2*B*···F3	0.94 (3)	2.04 (3)	2.961 (2)	167 (3)
N2—H2*C*···F4	0.83 (3)	2.02 (3)	2.849 (2)	172 (3)
N3—H3*A*···F1^iv^	0.88 (3)	2.57 (3)	3.274 (2)	138 (3)
N3—H3*A*···F2^viii^	0.88 (3)	2.42 (3)	3.223 (2)	153 (3)
N3—H3*B*···F3^vii^	0.79 (3)	2.61 (3)	3.345 (3)	157 (3)
N3—H3*C*···F1^i^	0.91 (3)	2.39 (3)	3.279 (3)	167 (3)
N4—H4*A*···F2	0.90 (3)	2.04 (3)	2.930 (2)	170 (2)
N4—H4*B*···F4^ix^	0.84 (3)	1.97 (3)	2.7955 (19)	171 (3)
N4—H4*C*···F1^iv^	0.79 (3)	2.33 (3)	3.045 (2)	151 (3)
N5—H5*A*···F4	0.96 (3)	1.90 (3)	2.8305 (19)	160 (3)
N5—H5*B*···F4^iii^	0.95 (3)	1.93 (3)	2.882 (2)	173 (2)
N5—H5*C*···F2^ii^	0.93 (3)	2.09 (3)	2.999 (2)	163 (3)

Symmetry codes: (i) −*x*+1, −*y*, −*z*+1; (ii) *x*, −*y*+1/2, *z*+1/2; (iii) *x*, −*y*+1/2, *z*−1/2; (iv) *x*−1, *y*, *z*; (v) *x*−1, −*y*+1/2, *z*−1/2; (vi) *x*, *y*, *z*+1; (vii) −*x*+1, −*y*, −*z*+2; (viii) −*x*, −*y*, −*z*+1; (ix) *x*−1, *y*, *z*−1.
